# Decoding Dengue: A Global Perspective, History, Role, and Challenges

**DOI:** 10.3390/pathogens14090954

**Published:** 2025-09-22

**Authors:** Flora Miranda Ulgheri, Bruno Gaia Bernardes, Marcelo Lancellotti

**Affiliations:** Biotechnology Laboratory—LABIOTEC, Faculty of Pharmaceutical Sciences, UNICAMP—State University of Campinas, Campinas 13083-852, SP, Brazil; f215994@dac.unicamp.br (F.M.U.); brunogaiabernardes@gmail.com (B.G.B.)

**Keywords:** DENV, dengue, *Flaviviridae*, public health, epidemiology

## Abstract

Dengue, caused by the dengue virus (DENV), is rapidly expanding its geographical footprint, with increasing incidence not only in over 100 endemic countries in the southern hemisphere but also with more autochthonous transmissions now reported in the northern hemisphere, including regions of Europe and the United States. The clinical presentation of DENV infection ranges from mild febrile illness to severe and potentially fatal conditions, such as dengue hemorrhagic fever (DHF), dengue shock syndrome (DSS), and diverse neurological complications. While vaccine development efforts are underway, significant challenges remain, underscoring the urgent need for a deeper understanding of the virus. This urgency is particularly palpable in Brazil, which has faced an unprecedented surge in dengue cases during the 2024–2025 period. The country has recorded an alarmingly high number of infections and related deaths, stretching its public health infrastructure and highlighting the complex interplay of climate change, urbanization, and viral dynamics in disease propagation. This review provides a global perspective on dengue, systematically exploring its history, morphology, viral cycle, pathogenesis, and epidemiology. By integrating these critical aspects, this article aims to identify pivotal knowledge gaps and guide future research directions essential for developing improved public health interventions against this complex and evolving disease.

## 1. Introduction

Dengue is an acute febrile viral illness transmitted to humans through the bite of an infected mosquito. The dengue virus (DENV) is an enveloped, positive-sense, single-stranded RNA virus, comprising four distinct serotypes: DENV-1, DENV-2, DENV-3, and DENV-4. Infection can manifest as a spectrum of outcomes, from asymptomatic cases to severe forms like dengue hemorrhagic fever (DHF) and dengue shock syndrome (DSS). As the most significant arboviral disease globally, more than half of the world’s population is at risk, with an estimated 100 to 400 million infections occurring annually and 136 countries or territories having reported autochthonous dengue transmission [1,2] Notably, dengue disproportionately impacts the southern hemisphere. The primary vectors of the disease worldwide are the *Aedes aegypti* and *Aedes albopictus* mosquitoes [1].

A significant concern arises from the fact that the four viral serotypes do not confer cross-protection. This can lead to antibody-dependent enhancement (ADE) [2], a phenomenon where heterologous reinfection increases the likelihood of developing a more severe form of the disease. Given that epidemic outbreaks can involve multiple concomitant serotypes, ADE poses a substantial public health challenge [3,4,5].

In this context, the development and widespread availability of a DENV vaccine are crucial for global public health. Currently, only one tetravalent vaccine (offering protection against all four serotypes) is approved for use in various parts of the world. Additionally, numerous other vaccine formulations are under development or have been developed, employing diverse technologies such as attenuated, inactivated, recombinant subunit, viral vector, and DNA vaccines. To prevent ADE, all these formulations must effectively protect against all four serotypes and provide comparable levels of protection for each.

Accordingly, this review aims to compile and synthesize current knowledge concerning the dengue virus, with a particular focus on its morphology, genomic structure, infection mechanisms, diagnostic methodologies, epidemiological patterns, and available vaccines. Our objective is to contribute to a more comprehensive understanding of this pathogen, which continues to represent a significant global public health burden.

## 2. The Dengue Virus

### 2.1. Viral Structure

**Figure 1:**
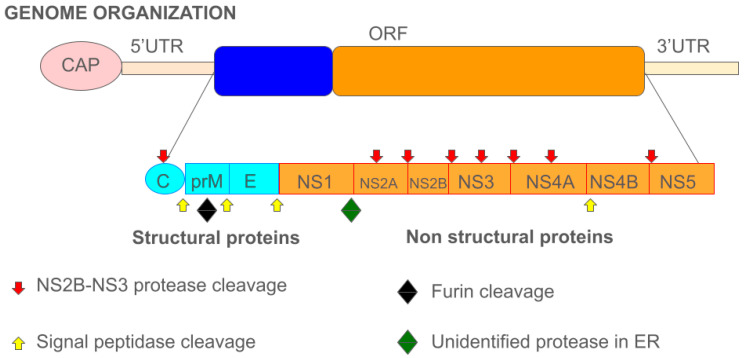
Dengue virus genome. Schematic representation of the *Orthoflavivirus denguei* genome in the 5′-3′ orientation, illustrating the segmentation and organization of its structural proteins (depicted in blue) and non-structural proteins (depicted in orange). Blue arrows pointing in four directions indicate cleavage sites recognized by furin; red arrows denote cleavage sites targeted by the NS2B-NS3 protease complex; yellow arrows represent cleavage sites processed by signal peptidases; and the green diamond marks the cleavage site mediated by an unidentified protease located in the endoplasmic reticulum [10].

The dengue virus, scientifically known as *Orthoflavivirus denguei*, is an arbovirus (arthropod-borne virus) belonging to the *Flaviviridae* family and the *Orthoflavivirus* genus [6,7]. It is an enveloped virus with an icosahedral shape, measuring between 40 and 60 nm in diameter, depending on its maturation state [1,2,8,9]. Its genome is a single-stranded, positive-sense RNA of approximately 10.7 kilobases. This genome is translated into a single polyprotein that is subsequently cleaved into three structural proteins—capsid (C), pre-membrane or membrane (prM/M), and envelope (E)—and seven non-structural proteins: NS1, NS2A, NS2B, NS3, NS4A, NS4B, and NS5 (Figure 1) [6]. The genes encoding the structural proteins are located at the 5′ end of the genome, while those for the non-structural proteins are at the 3′ end.

All three structural proteins possess well-defined functions. The DENV capsid is a simple protein primarily responsible for safeguarding the viral genome by aggregating its RNA during assembly and facilitating genome release during the infection process [3]. The membrane is formed during a maturation process: the immature virion, initially containing envelope trimers associated with prM proteins, undergoes cleavage of the “pr” peptide by the cellular protease furin. This leaves only the “M” membrane as a transmembrane protein beneath the E protein in the mature particle [11]. This protein plays a crucial role in viral entry into the host cell, as the fusion of the virion membrane with the host cell membrane is essential for infection in enveloped viruses [3,6].

Finally, the envelope protein protects the genomic RNA and also binds to the host cell membrane during entry and uncoating. Generally, DENV’s non-structural proteins are involved in viral replication and dissemination, though they have diverse individual roles [12]. The first non-structural protein, NS1, is a glycoprotein initially expressed as a monomer in infected cells. It then forms homodimers with organelle and cell membranes after undergoing post-translational modification in the endoplasmic reticulum (ER) [13]. Studies suggest that NS1 may cause endothelial dysfunction, leading to vascular leakage in more severe cases of infection [14]. Furthermore, NS1 can disrupt the coagulation process [11].

The period of viremia in dengue is closely linked to the presence of NS1 in the patient’s serum, being detectable at the onset of the febrile phase. This makes NS1 a valuable target for rapid dengue diagnostic tests, especially as other tests face challenges like difficulty in acute phase diagnosis or cross-reactive results with other flaviviruses due to shared antigenic epitopes [15,16].

NS2A, along with NS2B, NS4A, and NS4B, is a membrane protein. Like the others mentioned, it contains transmembrane helices critical for its localization within the cell membrane. This protein interacts with RNA and various other proteins to perform functions in viral replication and pathogenesis, making it a key component of the membrane-bound replication complex [14,17].

A 2015 study revealed that specific mutations within NS2A residues did not impede viral RNA replication, yet they critically prevented the formation of infectious viral particles [18]. This finding highlights NS2A’s essential role beyond replication. Furthermore, NS2A is directly involved in viral assembly and possesses the capacity to autonomously counteract the host’s interferon-alpha/beta (IFNα/β) response, which consequently impacts viral replication. Interestingly, NS2A can also induce apoptosis in non-functional cells [18,19].

As previously noted, NS2B is also a membrane protein. This relatively small protein is primarily recognized for its regulatory role as a cofactor for the NS3 protease, forming a functional complex through its hydrophilic regions. Beyond this well-established interaction, NS2B participates in various molecular interactions crucial for both viral replication and assembly [20].

Working in concert with NS2B, NS3 forms a complex responsible for the precise cleavage of the endoplasmic reticulum (ER) membrane polyprotein on its cytoplasmic side. This proteolytic activity is vital for processing the viral polyprotein into individual functional proteins, specifically at the C/prM, NS2A/NS2B, NS2B/NS3, NS3/NS4A, and NS4B/NS5 junctions. Additionally, other studies suggest that NS3 contributes to viral particle assembly through interactions with other non-structural proteins, independent of its enzymatic functions [18,21,22].

The membrane proteins NS4A and NS4B are interconnected via a peptide segment referred to as 2K. The initial cleavage event, occurring at the NS4A-2K junction, is facilitated by the NS2B-NS3 protease and is essential for enabling the subsequent proteolytic processing between 2K and NS4B [23].

Both proteins perform multiple functions in viral replication and virus–host cell interactions. These include regulating the formation of viral replication complexes via NS4A’s interaction with cellular vimentin; inducing autophagy (mediated by NS4A) to promote replication while preventing cell death; dissociating NS3 from single-stranded RNA (mediated by NS4B); modulating viral replication through the interaction of both (NS4A/B) with NS1; and inhibiting IFN signaling [24,25].

Finally, NS5 is the largest of the seven non-structural proteins and is the most conserved among the four dengue serotypes, showing approximately 70% sequence similarity [26]. It possesses two primary functions: RNA-dependent RNA polymerase (RdRp) activity, located in the C-terminal region, which is essential for viral replication; and RNA methyltransferase (MTase) activity, located in the N-terminal region, required for RNA capping during polyprotein translation. Beyond these main functions, NS5 can also negatively regulate the host cell’s IFN response [27].

### 2.2. Viral Cycle

Like other arboviruses, the dengue virus requires a host for replication and a vector for transmission. In the case of dengue, the primary vectors are the mosquitoes *Aedes aegypti* and *Aedes albopictus* [28,29]. Also, similar to other arboviruses, dengue originated in non-human primates (NHPs), which typically show no signs of viral infection. This transmission cycle between susceptible NHPs and *Aedes* mosquitoes is known as the sylvatic transmission cycle. The dengue virus likely emerged from sylvatic cycles in Asian forests, which have also been observed in African forests [30,31].

Dengue’s urban cycle, also referred to as the epidemic cycle, involves transmission between humans and *Aedes* mosquitoes. Both the sylvatic and urban cycles are categorized as horizontal transmission cycles. Beyond horizontal transmission, it is known that infected mosquitoes (both male and female) can transmit DENV to a portion of their offspring through a process called vertical transmission [32,33].

**Figure 2:**
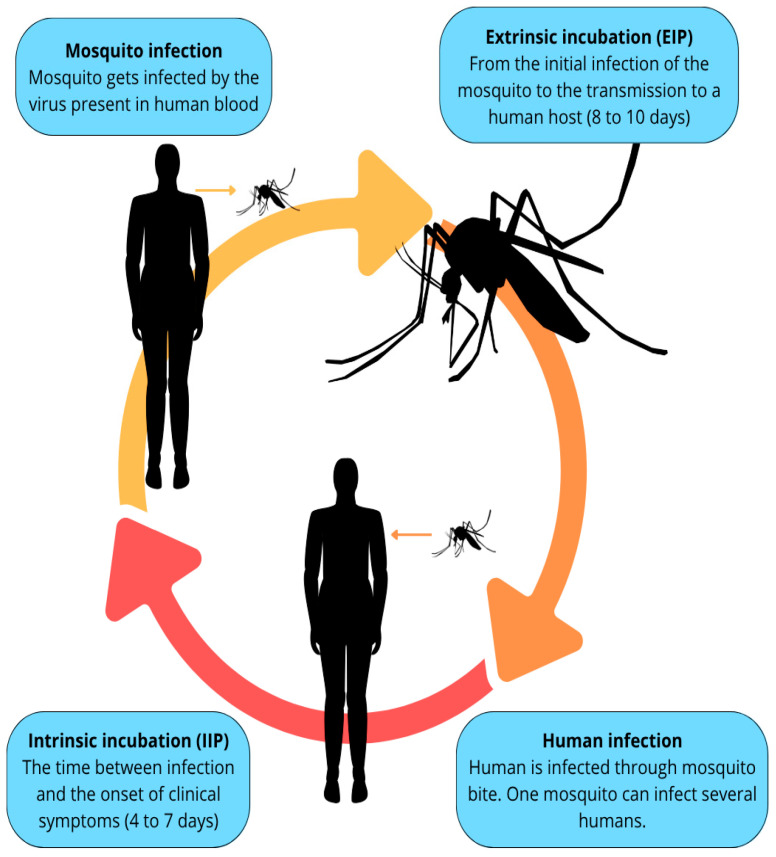
Viral replication cycle. In an infected mosquito, the virus undergoes the extrinsic incubation period (EIP), which spans from the initial infection of the mosquito to the transmission of the virus to a human host, typically lasting between 8 and 10 days. Following this period, the virus is transmitted to humans via the bite of the vector. Once in the human host, the virus enters the intrinsic incubation period (IIP), defined as the time between infection and the onset of clinical symptoms, generally ranging from 4 to 7 days [5].

The incubation periods of the virus can be either extrinsic or intrinsic. The extrinsic incubation period (EIP) is the time from when the mosquito takes a blood meal from an infected source until it becomes infectious. The intrinsic incubation period (IIP) is the time between human infection and the onset of disease symptoms [7,34] (Figure 2).

During the EIP, once the mosquito ingests an infected blood meal, the dengue virus enters its system. Initially, it infects the midgut epithelial cells via receptor-mediated endocytosis, successfully crossing the midgut infection barrier (MIB). The fact that DENV is transmitted via *Aedes* mosquitoes as vectors leads to the hypothesis that the virus’s transmission cycle involves a unique interaction with both the human host and the surrounding environmental factors [20,34].

The clathrin-coated endosome then triggers conformational changes in the E protein, facilitating the fusion of the viral envelope with the endosomal membrane, which leads to the release of the nucleocapsid into the cytoplasm [35]. At this stage, the viral RNA is translated into a polyprotein. This polyprotein is then directed to the endoplasmic reticulum, where it is processed before the initiation of RNA replication, mediated by non-structural proteins [36,37].

If this replication process is successful, the newly formed virions must traverse the basal lamina surrounding the midgut, also known as the midgut escape barrier (MEB), to reach the hemocoel. The virus then travels through the hemolymph and hemocytes to reach secondary tissues, including muscles, neural tissues, and salivary glands [38,39].

After crossing the salivary gland infection barrier (SGIB) and replicating within the cytoplasm and apical region of acinar cells, the final step for DENV is to reach the salivary duct. This duct is considered the last barrier (the salivary gland escape barrier or SGEB) before the virus can be present in the insect’s saliva and begin infecting vertebrate hosts [38]. The incubation period in the mosquito typically lasts 8 to 10 days (though some literature indicates up to 12 days), after which the insect can transmit the virus for the remainder of its life [40].

In humans, the incubation period averages 4 to 7 days. The initial cells infected by the virus are dendritic cells in the skin, specifically Langerhans cells, and macrophages residing in the skin. Viral entry is facilitated by receptors and co-receptors on the host cell surface and the viral E protein. The M protein also assists in binding to the host cell receptor. After entering the cell, the virus is internalized within an endosome. Following conformational changes within this structure, the viral envelope fuses with the endosomal membrane, releasing the nucleocapsid into the cellular cytoplasm [41,42].

The viral genome dissociates from the C protein and moves to the rough endoplasmic reticulum, where it can act as mRNA for protein synthesis and serve as a template for viral replication. After viral replication and the assembly of viral proteins within the host cell, the virus undergoes a maturation process as it moves from the Golgi complex to the trans-Golgi network (TGN). During this maturation, the prM protein is cleaved by the furin protease. Once mature, the virus exits the TGN, moves to the cytoplasm, and is finally exocytosed from the host cell’s plasma membrane [41].

After replicating in the skin’s dendritic cells, these infected cells come into contact with lymph nodes, leading to the infection’s spread to macrophages and monocytes. This subsequently results in viremia due to the virus’s interaction with draining and distant lymph nodes [42]. In addition to the previously mentioned cells and tissues, DENV can be identified in tissues such as the bladder, kidneys, lungs, and liver. Viremia can be detected for 10 to 12 days, beginning 24 to 48 h before the appearance of clinical symptoms [41,42].

### 2.3. Infection and ADE

**Figure 3:**
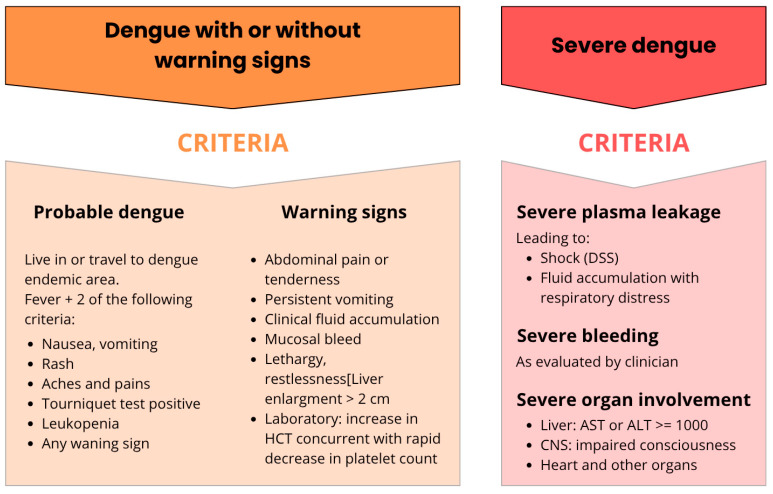
Warning signs associated with different clinical presentations of dengue. Indicators of probable dengue infection and its various warning signs, as well as diagnostic criteria for severe dengue, which may involve severe plasma leakage, severe hemorrhage, and severe organ impairment [45].

Dengue fever (DF) cases can range from mild, undifferentiated febrile illness to more severe forms like dengue hemorrhagic fever (DHF) or dengue shock syndrome (DSS) [43]. Since 2009, the World Health Organization (WHO) has classified cases according to their severity levels: dengue without warning signs, dengue with warning signs, and severe dengue [44,45] (Figure 3).

According to the current WHO classification, dengue warning signs include abdominal pain or tenderness, persistent vomiting, clinical fluid accumulation, mucosal bleeding, lethargy, restlessness, and liver enlargement. Severe dengue cases encompass severe plasma leakage leading to shock (DSS) and fluid accumulation with respiratory distress, severe bleeding, and severe organ involvement (such as the liver, heart, and others). Several individual risk factors contribute to varying degrees of disease severity, including age, ethnicity, secondary infection, and chronic diseases like bronchial asthma and diabetes mellitus [46].

Beyond the aforementioned clinical manifestations, DENV infection can lead to neurological complications, broadly categorized into three groups:

- Neurotropic effects: encephalitis [47], meningitis [40], myositis [48], rhabdomyolysis [49], and myelitis [48].
- Systemic complications: encephalopathy [9], hemorrhagic and ischemic stroke [48,50,51], hypokalemic paralysis [51], and papilledema [52].
- Post-infection effects: acute disseminated encephalomyelitis (ADEM) [53], encephalomyelitis [48], myelitis [54], neuromyelitis optica [55], optic neuritis [56], Guillain–Barré syndrome [57], possible Miller–Fisher syndrome [58], phrenic neuropathy [59], long thoracic neuropathy [60], oculomotor paralysis [61], maculopathy [60], and fatigue syndrome [54].

These neurological effects are more broadly associated with serotypes 2 and 3. Although DENV was initially considered a non-neurotropic virus, the detection of dengue antigen in the brain ultimately led to the conclusion of neuroinvasion [48].

Host innate immune system cells, such as dendritic cells, macrophages, and monocytes, are the first line of defense against DENV infection through pattern recognition receptors (PRRs). The host’s antiviral state is triggered by PRR recognition, which induces the production of cytokines and chemokines [62].

Type I IFN responses are induced by the activation of certain receptors after DENV recognition. Type I IFN production prevents other monocytes from being infected by the virus, and the binding of these cytokines to IFNα/β receptors on the surface of infected or nearby cells enhances the host’s antiviral response. The complement system also plays a significant role by inducing lysis, recruiting phagocytes, and promoting inflammation [63]. During the early stages of a primary DENV infection, anti-dengue IgM levels begin to rise between 3 and 5 days after the onset of the febrile stage. They decline over the subsequent 2 to 3 months, being replaced by IgG, which remains detectable for life. IgG provides protection against reinfection by the same serotype but not against different serotypes, a phenomenon known as heterotypic immunity [42].

In the case of reinfection with a different serotype, a phenomenon initially described by Halstead and O’Rourke in 1977, known as antibody-dependent enhancement (ADE), can occur. This phenomenon was observed and studied to explain the increased disease severity often seen in secondary DENV infections [64]. There are two general classifications for ADE: extrinsic and intrinsic. The former enhances the entry of viral particles into immune cells, whereas the latter involves modulation of the immune response, thereby creating an intracellular environment conducive to viral replication [65].

Non-neutralizing antibodies, which constitute the majority, are directed against various antigens present in the E and prM proteins. In a secondary infection where specific neutralizing antibodies for the infecting serotype are absent, these non-specific antibodies facilitate the entry of the dengue virus into Fc receptor-bearing cells, such as macrophages and monocytes, thereby increasing viral replication. In essence, virus neutralization mediated by antibody binding is insufficient to combat the infection, yet significant enough to internalize opsonized viruses, leading to a “false-neutralization” phenomenon [64,66].

The DENV prM protein is of particular interest in ADE-related studies. Anti-prM antibodies exhibit low neutralization rates, remaining between 30% and 50% even at high concentrations. Since prM is typically lost during maturation, mature viruses lacking prM are not neutralized. Additionally, partially mature viruses may contain enough prM to bind antibodies but not enough to result in neutralization. Furthermore, ADE presents an additional challenge in the development of an effective antiviral dengue vaccine, a topic that will be discussed later [35,64,67,68,69].

In June 2025, the World Health Organization released new guidelines on the management of arboviral diseases in a manual entitled *Integrated Guidelines on the Clinical Management of Arboviral Diseases*. The document provides recommendations for the clinical management of the four most widely distributed arboviruses worldwide—dengue, chikungunya, Zika, and yellow fever [70].

It is important to emphasize that the previously available guidelines were primarily focused on the Americas. Consequently, the development of more comprehensive standards was necessary to address other regions with endemic transmission as well as areas at risk of viral introduction [70].

**Figure 4:**
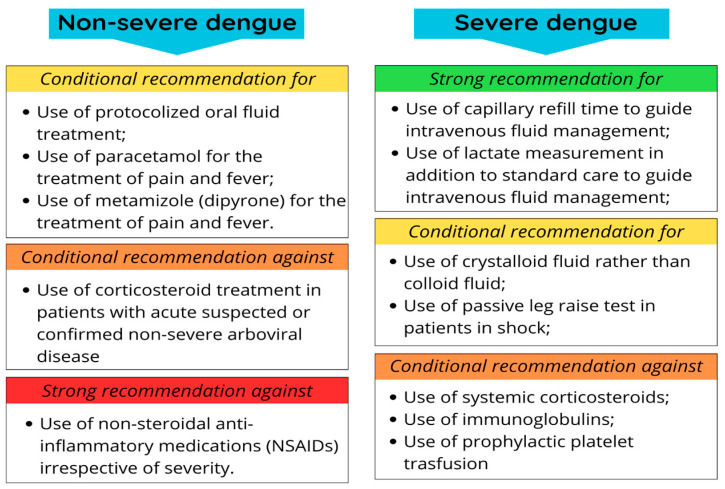
WHO 2025 recommendations for patients with non-severe and severe dengue. Clinical recommendations for or against dengue cases, more detailed in the WHO guidelines for clinical management of arboviral diseases [70].

For both patients with non-severe and severe dengue, the WHO provides recommendations divided into four categories (Figure 4): strong recommendation with low-certainty evidence; conditional recommendation with low-certainty evidence; conditional recommendation against with very low-certainty evidence; and strong recommendation against with low-certainty evidence [70].

It is important to note that these recommendations may or may not be applicable to other arboviral diseases covered in the guidelines, such as Zika, chikungunya, and yellow fever [70].

### 2.4. Diagnosis

To diagnose dengue, it is essential to either isolate the virus or detect its genome. Existing diagnostic tests fall into two main categories: those that confirm the presence of the virus (evaluating viral antigens and the viral genome) and those that detect the host’s immune response to infection (assessing antibodies) [71,72].

The history of serological tests for dengue began in 1950 with the discovery that dengue—and arboviruses in general—can agglutinate certain types of erythrocytes [71]. The hemagglutination inhibition (HI) assay is a simple, rapid technique used to determine the relative amount of virus in a sample. This test relies on the ability of DENV antibodies to prevent agglutination. However, it often struggles to differentiate dengue infections from those caused by similar flaviviruses, such as Japanese encephalitis virus and West Nile virus [73,74,75].

The plaque reduction neutralization test (PRNT), a method developed in 1967 by Russell et al., offers superior specificity in diagnosing dengue by distinguishing its antibodies from those of other flaviviruses. Although it is the most widely used test for immunity studies, it is labor-intensive and time-consuming, making it less common for routine DENV infection diagnosis [76,77,78,79].

The MAC-ELISA test (Immunoglobulin M Antibody-Capture Enzyme Linked ImmunoSorbent Assay) is an easily reproducible test widely used for diagnosing viral pathogens [71]. This test measures the increase in dengue-specific IgM levels in primary and secondary infections by analyzing the patient’s serum [80]. An Antidengue IgG MAC-ELISA test also exists, but it has lower specificity due to cross-reactivity [47].

Beyond these methods, the ELISA NS1 test is currently used to detect the presence of the dengue virus’s non-structural NS1 protein. This method utilizes rabbit polyclonal antibodies as capture antibodies and rabbit monoclonal antibodies as detection antibodies [81]. The indirect fluorescent antibody test, developed in 1973, detects dengue-specific IgM and IgG using fluorescent antibodies. This method is primarily used in research laboratories, not for routine diagnostics, as it is low-sensitivity and non-automatable [82]. The Dot-Blot test is a rapid diagnostic test, typically taking around 4 to 6 h, requiring minimal expertise and laboratory materials. This test detects both dengue IgM and IgG, allowing it to differentiate primary from secondary infections [71].

The second group of laboratory diagnostics includes tests for virus identification and isolation. These tests began in 1942 when the dengue virus was first isolated. In this initial event, blood from infected patients was intracerebrally inoculated into suckling white mice. Since then, the technique has become a standardized method for generating serotype-specific dengue antigens. The drawback of this test is that it is not only complex and time-consuming but also requires a stable supply of suckling mice [71,83].

Due to these issues, other viral isolation techniques were developed, such as mosquito inoculation. This technique, developed in the 1970s, uses mosquitoes like *Toxorhynchites splendens* and *Aedes* mosquitoes. *Toxorhynchites* mosquitoes are larger and easier to inoculate with human serum than *Aedes* mosquitoes and do not feed on blood, preventing virus transmission. Also, there are many cell lines available to inoculate the virus into cell cultures, generally divided into insect cells and mammalian cells [71].

Since cross-reaction frequently occurs between dengue serotypes, there are few methods to determine the infecting virus serotype in a patient’s serum using antibodies. One procedure involves isolating the virus through the previously mentioned techniques and subsequently identifying the serotype using monoclonal antibodies [71].

Beyond serology tests and virus identification/isolation, genome-based assays exist. Unlike IgM- and IgG-based methods, these can directly identify the dengue virus. The first of these is RT-PCR, a technique involving DNA amplification to produce cDNA through a reverse transcriptase reaction from an RNA fragment. It is currently one of the methods used for detecting and serotyping dengue [81,84,85]. The RT-PCR method is highly sensitive, capable of detecting the virus even in the early stages of the disease, and can also be used for virus detection in mosquitoes [86,87]. A limitation of this assay is the need for specific equipment such as an ultracentrifuge, thermocycler, and electrophoresis [88].

Another technique used is nucleic acid sequence-based amplification (NASBA), which involves the use of electrochemiluminescence to detect mRNA. RNA amplification by this method is performed directly using primers and capture probes at isothermal temperatures [71]. One more technique for DENV detection is fluorogenic probe-based 5′ exonuclease (TaqMan). In this technique, a fluorescent probe hybridizes with the target cDNA sequence, and a fluorescent signal is released through the 5′-3′ exonuclease activity of Taq DNA polymerase. Its average duration is 4 h, and it offers advantages over RT-PCR, such as eliminating the need for electrophoresis and reducing contamination risk, as the product is detected without opening the reaction tube [89,90].

Similar to the new guidelines for the clinical management of dengue, in 2025 the WHO also drafted a guide focused on laboratory testing for the dengue virus. This document outlines current evidence related to diagnostics and laboratory assays, while also providing recommendations for institutions such as laboratories, public health officials, clinicians, and other stakeholders [91].

### 2.5. History and Epidemiology

The earliest recorded instance of an illness with symptoms consistent with dengue dates back to 265–420 AD, documented in a Chinese encyclopedia later edited in 610 and again in 992 AD. Epidemics resembling dengue were reported in the West Indies in 1635 and Central America in 1699. Additional potential outbreaks occurred in 1779 in Jakarta (Indonesia) and Cairo (Egypt). However, it was in 1780 that a dengue outbreak was retrospectively confirmed in North America, specifically in Philadelphia [92].

The four DENV serotypes are thought to have originated from similar ecological cycles involving sylvatic transmission between non-human primates and mosquitoes, as well as urban cycles between humans and mosquitoes. Human migration and trade facilitated the virus’s spread to villages and cities across Asia, transmitted by *Aedes* and *Stegomyia* mosquitoes. Some authors, like Ehrenkranz et al., have speculated that the virus originated on the African continent and was subsequently disseminated via the transatlantic slave trade [93]. An alternative hypothesis proposed by Halstead suggests that the dengue virus may have emerged from a sylvatic cycle involving primates and mosquitoes in the Malaysian Peninsula [64,94].

The four serotypes were initially identified in different geographical regions. DENV-1 was first detected in Hawaii, New Guinea, and India, with the Hawaiian strain (Haw-DENV-1) becoming the reference strain. DENV-2 was also isolated in New Guinea, and its reference strain is the New Guinea C strain (NGC-DENV-2). Both DENV-3 (Philippines/H87/1956) and DENV-4 (Philippines/H241/1956) were isolated in 1956 during a dengue outbreak in Manila, the capital of the Philippines [95].

Beyond the four recognized serotypes, in 2007, a 37-year-old patient was admitted to a hospital in Sarawak, Malaysia, with what was initially presumed to be a typical case of dengue caused by DENV-4. However, upon isolation and genomic analysis, the virus was found to differ genetically from DENV-4 while sharing some similarities with DENV-2 [92].

Initially considered a variant of DENV-4, the virus was later tested in rhesus monkeys previously infected with the four known serotypes. These monkeys produced significantly different antibodies, indicating that the virus was not a DENV-4 variant but rather a distinct fifth serotype, designated DENV-5 [92]. As with the other serotypes, DENV-5 is assumed to have circulated in Southeast Asian forests among non-human primates before spilling over into humans [96,97].

According to the World Health Organization, dengue is currently an endemic disease in over 100 countries across Southeast Asia, Africa, the eastern Mediterranean, the Americas, and the western Pacific, with its incidence having increased approximately eightfold since the year 2000 and 30-fold in the last 50 years [92,98].

#### 2.5.1. Southeast Asia

Since the 1960s, all four serotypes of the dengue virus (DENV) have been endemic to Southeast Asia, with established patterns of distribution persisting throughout the region [99]. Of the countries comprising this area, ten are considered dengue-endemic, placing approximately 1.3 billion individuals at risk of infection [100]. With the exception of North Korea, all countries in the region—also referred to as the Southeast Asian Region (SEAR)—experience recurrent dengue epidemics in a cyclical manner [101]. Among these, countries such as India and Sri Lanka are classified as hyperendemic. In the latter, dengue is believed to be the deadliest mosquito-borne disease nationwide [99,100]. In India, all four DENV serotypes are present and co-circulate with varying degrees of predominance. For instance, DENV-2 and DENV-3 are more prevalent in the southern region, while DENV-1 and DENV-2 predominate in the north, according to a study conducted in 2017 [100,102]. Currently, the virus is widespread throughout Indian territory, and certain areas report notably high mortality rates [102].

#### 2.5.2. Africa

On the African continent, the first confirmed dengue outbreaks occurred in Zanzibar in 1823 and 1870 [103]. Overall, all four serotypes of the virus are present across the continent, where they are responsible for causing sporadic outbreaks involving a single serotype or multiple serotypes in co-circulation [104]. The most prevalent serotype in Africa is DENV-2, followed by DENV-1. The subregions most affected—ranked by the frequency of recent outbreaks—are East Africa, West Africa, North Africa, and Central Africa. The least affected subregion in the past decade was North Africa; nevertheless, it has reported some cases of infection and may, in the future, become part of the endemic areas [105]. In 2023, only seven countries accounted for 98% of all reported dengue cases on the African continent: Burkina Faso, Chad, Ethiopia, Mali, Niger, Nigeria, and Sudan [106]. Additionally, international travel plays a significant role in the transmission of the virus from African countries to parts of Europe and Asia [105].

#### 2.5.3. Eastern Mediterranean

In the eastern Mediterranean region—which comprises approximately 24 countries across the Middle East and North Africa (MENA)—a total of 81 dengue outbreaks were reported between 1941 and 2015, all originating from just nine countries. The virus vector *Aedes aegypti* is present in 11 MENA countries, while *Aedes albopictus* is found in 7 countries, including Algeria, Palestine, and Syria—nations where the former species has not been reported. All four DENV serotypes have been identified in the region; however, outbreaks caused by DENV-1, DENV-2, and DENV-3 have been observed in countries surrounding the Red Sea—such as Djibouti, Egypt, Saudi Arabia, Yemen, Somalia, and Sudan—whereas DENV-4 has only been reported in Pakistan, alongside the other three serotypes [107]. It is important to note that multiple risk factors are associated with countries in this region, complicating the feasibility of comprehensive studies and contributing to the increased prevalence of the virus. These include humanitarian emergencies leading to weakened healthcare systems, high numbers of refugees, unplanned urbanization, climate change, and limited disease surveillance, among other factors [108].

#### 2.5.4. Americas

Dengue’s presence in the Americas is not new; indeed, the region has a long and often devastating history with this arboviral disease. Early records suggest the disease likely arrived with the transatlantic slave trade and European colonization, establishing a foothold in the Caribbean and tropical mainland long before its modern resurgence. The first major documented epidemic in the Americas occurred in Philadelphia in 1780, an event that retrospectively confirmed dengue’s capacity for widespread urban transmission in the western hemisphere [92]. Throughout the 19th and early 20th centuries, dengue outbreaks were sporadic but often severe, particularly in port cities and areas with high *Aedes aegypti* mosquito populations. However, widespread mosquito control efforts, particularly during the mid-20th century, led to a significant decline in dengue cases and even the eradication of *Aedes aegypti* in some areas [109].

Despite these earlier successes, the latter half of the 20th century witnessed a dramatic resurgence of dengue in the Americas. This comeback was fueled by several factors, including the re-infestation of *Aedes aegypti* due to relaxed vector control programs, rapid and unplanned urbanization, increased population density, and intensified regional and international travel [98,110]. The introduction of new DENV serotypes, often leading to sequential infections and a higher risk of severe disease, further exacerbated the situation.

Today, dengue outbreaks in the Americas typically follow 3- to 5-year cycles with distinct seasonal patterns, reflecting the influence of climate and vector dynamics. The scale of the current burden is immense. According to World Health Organization data, the first half of 2023 alone saw 2,997,097 dengue cases reported across the Americas, with a substantial 45% (1,348,234 cases) laboratory-confirmed. Brazil, a hyper-endemic country, bore the brunt of this impact, reporting 2,376,522 infections within that period [30].

The co-circulation of multiple DENV serotypes is a critical feature of the current epidemiological landscape in the Americas, elevating the risk of secondary, more severe infections due to antibody-dependent enhancement (ADE). In 2024, all four DENV serotypes (DENV-1, DENV-2, DENV-3, and DENV-4) were identified as circulating throughout the region. Countries like Brazil, Costa Rica, Honduras, and Mexico reported the presence of all four serotypes, while others such as Argentina and Puerto Rico saw DENV-1, DENV-2, and DENV-3. Guatemala reported DENV-2, DENV-3, and DENV-4, and French Guiana had DENV-2 and DENV-3. Bolivia and Paraguay experienced DENV-1 and DENV-2 circulation, and French territories including Guadeloupe, Martinique, Saint Barthélemy, and Saint Martin predominantly reported DENV-2 [30].

As of 2025, a total number of 3,662,751 suspected cases have been reported in the Americas in the epidemiological week 32. This number is 69% lower compared to the same period in 2024. Out of the total, 40% of the cases were laboratory confirmed. Equal to 2024, all four serotypes are circulating in the Region [110].

The significant increase in case numbers in recent years is a complex phenomenon driven by a confluence of intertwined factors [111]. These include:

- Changes in vector distribution: The geographic spread of *Aedes* mosquitoes continues to expand, reaching previously unaffected areas.
- Climate change: Rising temperatures and increased humidity create more favora-ble conditions for mosquito breeding and viral replication within the vector, shortening the extrinsic incubation period.
- Political and financial instability: Countries facing complex humanitarian crises often experience weakened healthcare systems and inadequate public health in-frastructure, hindering effective surveillance and control measures.
- Increased human mobility and tourism: The movement of people facilitates the rapid dissemination of the virus to new locations, leading to outbreaks in areas previously considered non-endemic.

Understanding this multifaceted historical and ongoing challenge is paramount for developing effective, sustainable strategies to control and prevent dengue in the Americas.

#### 2.5.5. Western Pacific

In the western Pacific region—which encompasses parts of Asia, Oceania, and the Pacific—the annual number of reported dengue cases rose from 430,023 in 22 countries in 2013 to 1,050,285 in 18 countries in 2019. All four serotypes have been identified in this region, though their prevalence varies by country. Among the countries reporting the highest number of DENV infections in 2019 were the Philippines (437,563 cases), Vietnam (320,702 cases), Malaysia (130,101 cases), Cambodia (68,597 cases), and Laos (44,250 cases). Collectively, these countries accounted for a total of 1,001,213 reported infections [112]. Several factors have been associated with the increase in case numbers in this region, including urbanization and population density growth, climate change, imported cases from international travel, and improved reporting to health authorities [112,113].

### 2.6. Vaccines

The development of a dengue vaccine faces a major challenge: an effective vaccine must provide protection against all four distinct serotypes of the virus while also eliciting balanced immune responses across them to prevent antibody-dependent enhancement (ADE) [114,115]. Vaccines that have been developed or are currently under investigation for dengue fall into five categories: live attenuated vaccines, inactivated vaccines, recombinant subunit vaccines, viral vector vaccines, and DNA vaccines [114].

Dengvaxia^®^ (CYD-TDV), developed by Sanofi/Pasteur (Paris, France), is a chimeric, tetravalent live attenuated vaccine. It is produced by replacing the prM and E proteins of Sanofi’s yellow fever vaccine (YFV 17D) with the corresponding prM and E proteins from the dengue virus. Its efficacy varies depending on factors such as the viral serotype, the age of the vaccine recipient, and their serological status [114,115,116]. At the moment this vaccine is no longer available and is not being commercialized by the manufacturer [109].

The vaccine developed by the National Institute of Allergy and Infectious Diseases (NIAID), known as TV003/TV005, is a tetravalent live attenuated virus (TLAV) vaccine. The selected target for this formulation was the 3′ untranslated region (3′ UTR) of flaviviruses, given its role in viral RNA replication [114]. This vaccine is composed of a mixture of four recombinant attenuated dengue viruses: rDEN1D30, rDEN2/4D30, rDEN3D30/31, and rDEN4D30. TV005 differs from TV003 by the inclusion of an additional inactivated viral component for DENV-2 [114,115,116].

TAK-003, also referred to as TDV or Qdenga^®^, is a tetravalent attenuated vaccine developed by the pharmaceutical company Takeda. It is based on recombinant DNA technology and consists of attenuated and chimeric viruses [114,115,117].

Other vaccines developed include TDEN F17/F19, which are live attenuated tetravalent vaccines composed of four DENV strains: DENV-1 (45AZ5), DENV-2 (S16803), DENV-3 (CH53489), and DENV-4 (341750). These viral strains were originally isolated from patients with natural dengue infections and propagated in C6/36 cells before being attenuated through serial passage in PDK cells. Sixteen formulations were synthesized in total; among them, formulations 13 and 14 were selected for further studies, and an additional formulation (F17pre) was developed to optimize the neutralizing antibody response. Of the three, F17 showed the most promising results in phase 2 clinical trials [115,118,119].

The purified and inactivated dengue vaccine (DPIV) is a two-dose tetravalent formulation. Its development began with a strain isolated from a patient, DENV-2 S16803, which was subsequently propagated in Vero cells. Given its effectiveness, the same process was applied to DENV-1 Westpac 74, DENV-3 CH53489, and DENV-4 TVP360 to produce a tetravalent vaccine using aluminum hydroxide as an adjuvant. However, after a series of studies, the vaccine was found to be unable to prevent DENV infection in rhesus monkeys immunized with the vaccine. Moreover, the observed increases in viremia, AST levels, IL-10, and IL-18 in the vaccinated animals suggest that the vaccination may have triggered antibody-dependent enhancement (ADE) [115,120,121].

The Tetravalent Dengue Virus Vaccine (TVDV) is a DNA-based vaccine administered in three doses. This vaccine utilizes the VR1012 plasmid to clone the pre-membrane (prM) and envelope (E) proteins from all four dengue virus serotypes, with these sequences derived from distinct strains [115,122].

For enhanced immunogenicity, the TVDV is co-administered with VAXFECTIN, a formulation composed of cationic and neutral lipids. VAXFECTIN functions as an adjuvant, meaning it significantly boosts antibody responses when combined with an antigen-encoding plasmid DNA [115]. The final tetravalent vaccine formulation is achieved by combining equal proportions of each monovalent plasmid, with each individual plasmid encoding the prM and E genes for one specific dengue serotype, all cloned into the VR1012 backbone [115,122].

The next vaccine, known as V180, was developed using recombinant prM glycoproteins and 80% of the E protein. Viral sequences from all four serotypes were amplified using RT-PCR from genetic constructs. For cloning, the pMttΔXho vector was employed, and the following strains were used: DENV-1 258848 and DENV-1 Thailand AHF82-80; DENV-2 PR159/S1; DENV-3 CH53489 and D3H87; and DENV-4 H241 and Dominica. It remains uncertain whether the levels of antibodies elicited are sufficient to provide long-lasting immunity without inducing ADE [115,118,123,124,125].

The DSV4, a virus-like particle (VLP)-based tetravalent vaccine, was designed by in-frame fusion of domain III of the envelope proteins (EDIII) from all four dengue serotypes with the hepatitis B surface antigen (HBsAg), in conjunction with an unfused S antigen, to promote VLP formation [115,126].

The E80-mRNA vaccine represents a recent advancement in vaccine development, utilizing a modified messenger RNA lipid nanoparticle (mRNA-LNP) platform. The complete vaccine construct is designed with a Cap1 sequence (N7mGpppAm) at its beginning. This is followed by a human IgE signal peptide sequence, which aids in protein secretion, and then the genetic sequences for the prM protein and the E80 protein [115,127].

The delivery system for this mRNA, the lipid nanoparticle (LNP) formulation, is composed of four distinct lipids. These are D-Lin-MC3-DMA, DSPC, cholesterol, and a PEG-lipid, present in a specific molar ratio of 50:10:38.5:1.5, respectively [115,127] (Table 1).

## 3. Conclusions

Dengue stands as a quintessential example of a rapidly escalating global health challenge, underscoring the urgent need for robust public health interventions against a broader spectrum of arboviral threats. The persistent and expanding prevalence of the dengue virus, exacerbated by the intricate dynamics of its transmission, the potential for severe clinical sequelae, and the complicating factor of antibody-dependent enhancement (ADE), collectively mandate sustained and intensified research endeavors. A thorough comprehension of the virus’s morphology, replication cycle, pathogenic mechanisms, and epidemiological patterns, as meticulously detailed in this review, forms the bedrock upon which effective and sustainable control strategies must be constructed. With this in mind, the availability of a compendium of up-to-date information on this disease remains constantly necessary in order to avoid knowledge gaps and prevent the dissemination of misinformation. The ongoing development of dengue vaccines offers a beacon of hope in the fight against this debilitating disease. However, the path to universal and highly efficacious vaccination is fraught with challenges. Further rigorous investigation is essential to address the inherent limitations of current vaccine candidates, particularly concerns regarding their efficacy across all serotypes and the potential for differential responses in seropositive versus seronegative individuals. Moreover, the dynamic nature of arboviruses, exemplified by the constant threat of emerging serotypes—such as the 5th serotype mentioned in some research—underscores the need for agile vaccine development platforms. These platforms must be capable of rapidly adapting to evolving viral strains. This continuous “arms race” between viral evolution and vaccine innovation highlights the critical importance of fundamental research into viral immunology and pathogenesis to stay ahead of the curve.

Concurrently, social science research is indispensable for understanding community perceptions, behaviors, and barriers to the adoption of preventive measures. Only through such a concerted, interconnected effort—spanning from the molecular laboratory to community engagement initiatives—can the world hope to effectively confront the escalating threat posed by arboviruses and safeguard global public health.

## Figures and Tables

**Table 1:** Dengue Vaccines.

Name	Year	Valence	Formulation	Developer/Manufacturer	Stage	Adjudicated
Dengvaxia (CYD-TDV)	2015	Tetravalent	YFV 17D and DENV-1-4 chimeric viruses	Sanofi Pasteur	Approved in Europe, United States and some Asian and Latin America countries	No
TV003/TV005	2003	Tetravalent	Three genetically attenuated viruses together with a chimeric virus	NIAID ^a^ (Bethesda, MD, USA) and Butantan (São Paulo, Brazil)	In vivo (stage IIIB)	No
QDenga (TAK-003)	2006	Tetravalent	Chimeric viruses from the attenuated DENV-2 PDK-53 virus	Takeda (Tokyo, Japan)	Approved in Europe, Brazil (?), Argentina, Indonesia and Thailand	No
TDEN	2017	Tetravalent	Viruses attenuated by multiple passages in PDK cells	WRAIR ^b^ (Silver Spring, MD, USA) and GlaxoSmithKline (London, UK)	In vivo (stage I–II)	No
DPIV	2012	Tetravalent	Inactivated and purified viruses Boosted with aluminum hydroxide AS01, AS03 or AS04	WRAIR ^b^, GlaxoSmithKline and FIOcruz (Rio de Janeiro, Brazil)	In vivo (stage I)	Yes
TVDV	2018	Tetravalent	DNA vaccine with the coding sequences for the prM and E proteins cloned into the VR1012 plasmid Adjuvanted with VAXFECTIN	U.S. AMRDC ^c^ (Fort Detrick, MD, USA), WRAIR, NMRC (Noida, India) and Vical (San Diego, CA, USA)	In vivo (animal and stage I)	Yes
V180	2018	Tetravalent	Recombinant proteins prM and 80% of E of the four serotypes Adjuvanted with ISCOMATRIX	Merck & Co. (Rahway, NJ, USA)	In vivo (stage I)	Yes
DSV4	2018	Tetravalent	DENV-1-4 EDIII VLPs with HBsAg	International Centre for Genetic Engineering and Biotechnology (New Delhi, India)	In vivo (animal)	No
E80-mRNA	2020	Tetravalent	mRNA expressing human IgE and the E80 protein contained in a lipid nanoparticle (LNP)	CAS laboratory of Molecular Virology and Immunology, Shanghai Pasteur Institute (Shanghai, China)	In vivo (animal)	No

^a^ National Institute of Allergy and Infectious Diseases; ^b^ Walter Reed Army Institute of Research; ^c^ U.S. Army Medical Research and Development Command. Vaccines currently under development for dengue. From left to right, the table presents the vaccine name, year of development, valence, a brief description of its formulation, the responsible developer, its stage of testing or licensure, and whether or not it is adjuvanted [115].
